# Transcriptional double-autorepression feedforward circuits act for multicellularity and nervous system development

**DOI:** 10.1186/1471-2164-12-228

**Published:** 2011-05-11

**Authors:** Hisakazu Iwama, Koji Murao, Hitomi Imachi, Toshihiko Ishida

**Affiliations:** 1 Life Science Research Center, Kagawa University, 1750-1 Ikenobe, Miki-cho, Kita-gun, Kagawa 761-0793, Japan; 2 Division of Endocrinology and Metabolism, Department of Internal Medicine, Faculty of Medicine, Kagawa University, 1750-1 Ikenobe, Miki-cho, Kita-gun, Kagawa 761-0793, Japan

## Abstract

**Background:**

The transcriptional regulatory network is considered to be built from a set of circuit patterns called network motifs. Experimental studies have provided instances where a feedforward circuit (FFC) appears with modification of autoregulation, but little is known systematically about such autoregulation-integrated FFCs. Therefore, we aimed to examine whether the autoregulation-integrated FFC is a network motif relevant to describing the human transcriptional regulatory systems, and explored the relationship of such network motifs with biological functions.

**Results:**

Based on human-mouse evolutionarily conserved transcription factor binding sites (TFBSs) in 76600 conserved blocks for 5169 genes, we compiled the human transcriptional connections into a matrix, and examined the number of FFC appearances in comparison with randomized networks. The results revealed that the configuration of autoregulation integrated in the FFC critically affects the abundance or avoidance of FFC appearances. In particular, an FFC comprising two repressors that are both autoregulated was revealed as a significant network motif, which we termed the double-autoregulation FFC (DAR-FFC). Interestingly, this network motif preferentially constitutes effecter transcriptional circuits with functions in cell-cell signaling and multicellular organization, and is particularly related to nervous system development.

**Conclusions:**

We have revealed that the configuration of autoregulation integrated in the FFCs is a critical factor for abundance or avoidance of the appearance of the FFCs. In particular, we have identified the DAR-FFC as a distinctive integrated network motif endowed with properties that are indispensable for forming the transcriptional regulatory circuits involved in multicellular organization and nervous system development. This is the first report showing that the DAR-FFC is a significant network motif.

## Background

It has been proposed that a complex system can be grasped using a small set of network motifs that recur significantly more often in the complex system than expected in its randomized network [1-5]. The transcriptional regulatory network of a cell is a complex system in which many transcription factor (TF) proteins turn gene expressions on and off according to spatiotemporal contexts. A feedforward circuit (FFC) comprising two regulators has systematically been shown to be a network motif in the transcriptional regulatory networks of *Escherichia coli *[1-5], yeast [2-6] and higher eukaryotes [4,7,8]. It is crucial to find an appropriate set of such basic network motifs to intelligibly describe the gene regulatory system.

One of the simplest network motifs is autoregulation, in which a regulator (TF) regulates the gene that encodes the regulator itself. An FFC is known to appear in some instances with modification of autoregulation [3,9,10], but no systematic studies on autoregulation-integrated FFCs have yet been carried out. Therefore, we aimed to elucidate whether the autoregulation-integrated FFC forms a distinct network motif relevant to describing the transcriptional network, and explored the relationships of this network motif with biological functions. For this purpose, we examined the influence of integrating autoregulation into the FFC on the number of FFC appearances by surveying the human transcriptional regulatory network.

**Figure 1:**
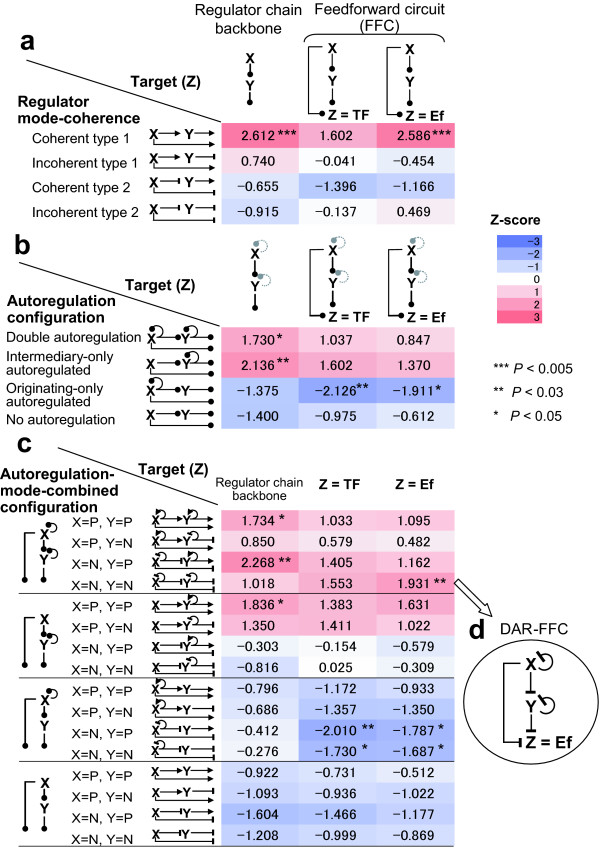
**Abundance or avoidance of various classes of FFCs**. Schematic diagrams of the representations of appearances of the individual circuits in the real human transcriptional network shown as heatmaps with Z scores (a negative value indicates underrepresentation and a positive value indicates overrepresentation) in comparison with the randomized networks. In each network motif, X denotes an originating regulator; Y, an intermediary regulator; Z, a target gene. The target gene is classified as either an effecter (Ef) or a transcription factor (TF). An arrow indicates positive regulation (also denoted as a P), a line terminated with -| indicates negative regulation (N), and a line terminated with a closed circle represents any mode of regulation. Each autoregulation is indicated with a semicircular line around the corresponding regulator symbol. A gray dashed semicircular line terminated with a gray closed circle represents either presence or absence of any mode of autoregulation. (**a**) The coherence classification shows marked overrepresentation of coherent type-1 FFCs targeting effecters. (**b**) A lack of autoregulation on the intermediary regulator critically decreases the abundance of FFCs. (**c**) The combined classification reveals a significant network motif and antimotifs. (**d**) The DAR-FFC is illustrated in a circle indicated from the corresponding Z score by an open arrow.

We compiled the human transcriptional connections into a connection matrix, based on the computationally identified human-mouse conserved TF binding sites (TFBSs) of 82 well-annotated TFs in 76600 conserved blocks located within the 8-kb upstream sequences of 5169 human genes that were stringent orthologs to mouse counterpart genes. Subsequently, we randomized the connection matrix in a degree-preserving manner [1-4,11,12], and obtained 1000 randomized matrices or networks. We then estimated the degree of overrepresentation or underrepresentation of the number of appearances for each circuit. The FFC we examined consisted of two regulators. The first regulator has effects on the second regulator such that the two regulators jointly regulate a target gene. The first regulator is termed the 'originating regulator' and the second regulator is termed the 'intermediary regulator'. We also examined the number of appearances of the 'regulator chain backbone', which represents a simple two-regulator one-way connection irrespective of the presence of a target gene common to the two regulators. To date, analyses of unicellular organisms have shown that (I) the FFC is a network motif [1-5], (II) the appearance of coherent FFCs outnumbers the appearance of incoherent FFCs [1,5, and please see the circuit graphs of 'coherent' and 'incoherent' FFCs in the left margin of Figure 1a] and (III) the coherent type-1 FFC composed of two activator regulators is the most abundant FFC [5]. Our dataset of human transcriptional connections also showed significantly overrepresented appearances of such FFCs, consistent with findings I, II and III in unicellular organisms.

In the present study, we further expanded the scope of network motifs to a category of small-scale integrated circuits by analyzing the autoregulation-integrated FFCs. In contrast to the conservativeness of the usage of simple FFC network motifs, our results revealed that the configuration of autoregulation integrated in the FFCs is a critical factor for abundance or avoidance of the appearance of the FFCs. In particular, we found that an FFC composed of two repressor regulators with autoregulation on each of them is a significant network motif. We report here that this novel network motif is preferentially involved in intercellular communication and multicellularity-oriented functions and particularly related to nervous system development.

## Results

### Overrepresentation of coherent type-1 FFCs

We first classified the FFCs into four classes based on the combinations of the modes (activator or repressor) of the originating and intermediary regulators [1,5] (Figure 1a). Following the FFC classification of Mangan and Alon [5], coherent and incoherent FFCs were further categorized as type 1 when the originating regulator was an activator and type 2 when it was a repressor. In addition, we separately evaluated the FFCs targeting TF genes and effecter (non-TF) genes because if an FFC targets a TF gene, the TF gene can again act as a node that further integrates other circuits but if an FFC targets an effecter gene, it is the terminal output of the FFC.

As a result, the total appearance of FFCs and the appearance of coherent FFCs were both significantly overrepresented in the real network, and the coherent FFCs outnumbered the incoherent FFCs (Table 1). In particular, the coherent type-1 FFC targeting an effecter gene was markedly overrepresented (*P *< 0.005, see the Methods) (Figure 1a). These overrepresentations were consistent with previous findings in *E. coli *[1,5] and yeast [5]. However, the degrees of representation of categories other than the coherent type 1 category did not show remarkable deviations from the random expectations.

**Table 1:** Statistical data for feedforward circuits and autoregulation in the real human transcriptional network in comparison with 1000 randomized networks.

		Appearance in the real network	Appearance in the randomized networks (mean ± s.d.*)	Z score	P*
Feedforward circuit (FFC)	Coherent	403774	378991 ± 13650	1.816	*P *≈ 0.035
	Incoherent	103468	105296 ± 7675	-0.238	*P *≈ 0.406
	Total	507242	484287 ± 13837	1.659	*P *< 0.05

Autoregulation		41	36.42 ± 2.95	1.551	*P *≈ 0.060

The mean, s.d. and *P *value of each circuit were computed based on the number of appearances in the 1000 randomized connection matrices (see the Methods).

### The configuration of autoregulation critically affects the FFC abundance

To examine the influence of autoregulation, we classified the FFCs according to the presence or absence of autoregulation on each regulator of an FFC without respect to the regulators' modes. This classification created four classes, which we termed (I) double-autoregulation, (II) intermediary-only autoregulated, (III) originating-only autoregulated and (IV) no autoregulation (Figure 1b). We found that categories III and IV, both of which have no autoregulation on the intermediary regulator, were consistently underrepresented, while in sharp contrast, categories I and II, both of which have autoregulation on the intermediary regulator, were overrepresented (Figure 1b).

Therefore, the absence of intermediary autoregulation was a hallmark for avoidance of FFC formation. In particular, the FFC composed of an autoregulated originating regulator and an autoregulation-less intermediary regulator was a significant antimotif common to TF and effecter target genes. Regarding the regulator chain backbone, autoregulation on the intermediary regulator was shown to be a significant factor for a higher frequency of appearance than expected in the random networks. These results demonstrated that the FFC, which was overrepresented as a whole, could be broken down into distinct classes that were heterogeneous in abundance or avoidance of the appearance of the FFC depending on the configuration of autoregulation.

### Autoregulation-and-mode combined classification of FFCs

Next, we combined the autoregulation-based classification with the classification based on the regulators' modes, and evaluated its applicability. This combined classification revealed two significant antimotifs of FFCs common to the TF and effecter target genes (Figure 1c). Both of these antimotifs were composed of an originating repressor regulator with autoregulation and an intermediary regulator without autoregulation irrespective of the mode. Since the degrees of representation of the backbone regulator chain for these motifs did not deviate much from the random expectations, the configuration of the autoregulated repressor with autoregulation-less intermediation is likely to have a specific disadvantage for feedforward synergistic control of the effecter.

### A novel network motif, the double-autorepression FFC (DAR-FFC)

The autoregulation-and-mode combined classification further revealed a significantly overrepresented network motif to which little attention has been paid. Specifically, this motif is the DAR-FFC that targets effecter genes in which both regulators are repressors with autoregulation (Figure 1d). Compared with the degree of representation of the backbone regulatory chain for this double-autorepression configuration, the DAR-FFC targeting an effecter showed a remarkable increase in its representation. This result indicates that the DAR-FFC has specific features that are advantageous for controlling effecter genes.

### Preferred functions of DAR-FFC-targeted effecter genes

To explore the functionalities in which DAR-FFCs play key roles, we assessed the types of biological activities preferred by DAR-FFCs. We conducted Gene Ontology (GO) [13] enrichment analyses on the effecter genes targeted by DAR-FFCs. Interestingly, we found that the most enriched molecular function of the DAR-FFC-targeted effecters was 'growth factor activity' (Table 2). The highly enriched functions of the DAR-FFC-targeted effecters were generally categorized into two functionalities, *i.e*. cell-cell signaling and auxiliary transcriptional regulation. The cell-cell signaling activities that were preferentially targeted by DAR-FFCs encompassed 'growth factor activity' and 'cytokine activity' as humoral cell-cell communication, 'voltage-gated ion channel activity' as a key role for neuronal transmission and 'extracellular matrix (ECM) structural constituent' together with 'integrin binding' as the right pair of ECM-mediated intercellular communications (Table 2). All of these functions represent major cell-cell communication activities in higher eukaryotes. On the other hand, the enrichment of auxiliary transcriptional regulator activity, such as 'transcriptional regulator activity' and 'transcription factor binding', also indicates an enhancement of the information integration capability at the transcriptional level, which corresponds to the increased demand for information processing potentially brought about by multicellularization.

**Table 2:** Preferred molecular functions of DAR-FFC-targeted effecters.

GO ID	GO molecular functions	*P*
GO:0008083	growth factor activity	1.47 × 10^-17^
GO:0003677	DNA binding	3.09 × 10^-16^
GO:0005244	voltage-gated ion channel activity	1.50 × 10^-13^
GO:0030528	transcription regulator activity	1.30 × 10^-12^
GO:0008134	transcription factor binding	2.00 × 10^-12^
GO:0030955	potassium ion binding	3.45 × 10^-12^
GO:0005249	voltage-gated potassium channel activity	4.99 × 10^-12^
GO:0005201	extracellular matrix structural constituent	5.70 × 10^-12^
GO:0005125	cytokine activity	1.36 × 10^-7^
GO:0005178	integrin binding	1.56 × 10^-6^
GO:0005509	calcium ion binding	5.40 × 10^-6^

This multicellularity-oriented involvement of DAR-FFCs was clearly demonstrated by GO enrichment analyses of biological processes in the DAR-FFC-targeted effecters, in which 'multicellular organismal development' topped the list with marked statistical significance (*P *≈ 5 × 10^-32^, see the Methods) (Table 3). The list subsequently included 'nervous system development', 'Wnt receptor signaling pathway' and 'axon guidance', indicating the particular importance of DAR-FFCs for nervous system development. Furthermore, 'transmembrane receptor protein tyrosine kinase signaling pathway', which contributes diverse intercellular communications, was also revealed as a biological process preferred by the DAR-FFC-targeted effecters.

**Table 3:** Preferred biological processes of DAR-FFC-targeted effecters.

GO ID	GO biological processes	*P*
GO:0007275	multicellular organismal development	5.31 × 10^-32^
GO:0007399	nervous system development	2.67 × 10^-17^
GO:0016055	Wnt receptor signaling pathway	3.20 × 10^-16^
GO:0007411	axon guidance	5.48 × 10^-12^
GO:0006355	regulation of transcription, DNA-dependent	3.05 × 10^-10^
GO:0006813	potassium ion transport	8.34 × 10^-10^
GO:0007156	homophilic cell adhesion	6.96 × 10^-8^
GO:0001525	angiogenesis	3.43 × 10^-7^
GO:0009887	organ morphogenesis	3.66 × 10^-7^
GO:0007169	transmembrane receptor protein tyrosine kinase signaling pathway	3.95 × 10^-7^
GO:0007155	cell adhesion	7.25 × 10^-7^

### DAR-FFC interlinks forming higher-order DAR-FFCs

**Figure 2:**
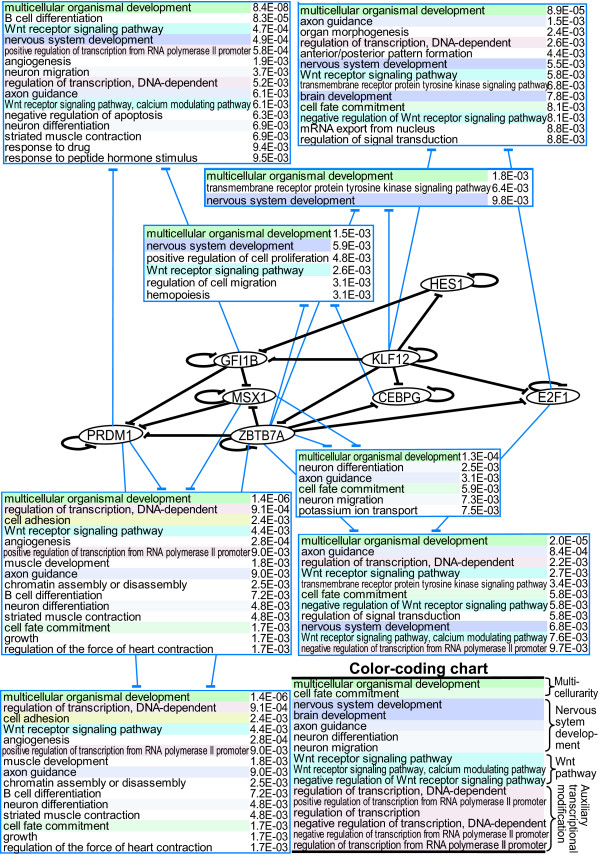
**Interlinks of DAR-FFCs and their preferred target gene functions**. Repressor TFs with autoregulation are designated by ovals. Each straight black line terminated with -| indicates a TF-TF connection for the DAR-FFC and each circular black line terminated with -| indicates autoregulation. A pair of thin blue lines indicates negative connections to the effecter gene repertoire targeted by the corresponding pair of TFs. The preferred GO 'biological processes' of the target effecters for each distinct TF-TF connection of the DAR-FFC are shown as a list at the end of a pair of blue lines. The terms in the lists are color-coded according to the categorization shown in the inset color-coding chart (bottom right).

The TFs that constitute DAR-FFCs in the real human transcriptional networks were found to be densely interconnected (Figure 2). All of these DAR-FFCs were shown to target effecter genes that preferentially contribute to 'multicellular organismal development' (Figure 2 andAdditional file 1). Notably, in 10 of the 13 DAR-FFCs, the biological process 'multicellular organismal development' topped the lists, followed by biological processes related to nervous system development (Figure 2 andAdditional file 1). These results suggest that the preferential involvement of DAR-FFCs in nervous system development is a general feature of DAR-FFCs.

**Figure 3:**
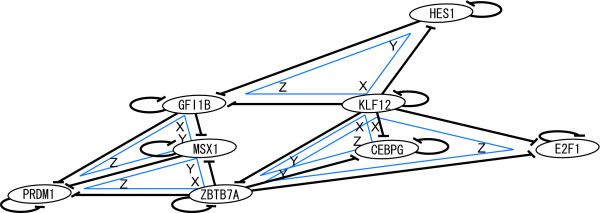
**Higher-order network structures of DAR-FFCs**. Repressor TFs with autoregulation are designated by ovals. Each straight black line terminated with -| indicates a TF-TF connection that constitutes a DAR-FFC and each circular black line terminated with -| indicates autoregulation. The higher-order network structures of the DAR-FFCs are individually depicted as blue triangles inside the corresponding TF-TF connections. The role assigned to a TF by each individual higher-order DAR-FFC is indicated with a symbol (X = originating regulator; Y = intermediary regulator; or Z = target) along the corresponding axis near the angle of the triangle. This schema illustrates that KLF12 always acts as an X (originating regulator) in all three higher-order DAR-FFCs in which it is involved, indicating that it may function as a 'higher-order originating regulator hub'.

One of the merits of considering the DAR-FFC as a unit is that it allows us to recognize a higher-order network structure. The TF-TF connections that constitute individual DAR-FFCs targeting effecter genes in turn form a higher-order DAR-FFC when a DAR-FFC's target gene is a TF that participates in the other DAR-FFC (Figure 3). For example, as shown in Figure 3, a higher-order DAR-FFC consists of two DAR-FFC TF-TF connections, *i.e*. one is MSX1-| PRDM1 and the other is ZBTB7A -| PRDM1. In this higher-order DAR-FFC, MSX1 is deemed to be an intermediary regulator and its feedforward path corresponds to the connection from ZBTB7A to PRDM1. Consequently, PRDM1 (a TF) is deemed to be a target gene. In the higher-order DAR-FFC, the important difference from the individual DAR-FFCs is the target. The target of a higher-order DAR-FFC is a TF, whereas the target of an individual DAR-FFC is a set of effecter genes. In the same way, Figure 3 demonstrates that MSX1 functions as an intermediary regulator of the feedforward path of GFI1B-| PRDM1.

As a result, we identified five higher-order DAR-FFCs in 13 TF-TF connections of the DAR-FFCs. This densely connected feature of the higher-order DAR-FFCs coincided with the results of motif representation analyses in the autoregulation-and-mode combined classification in which the DAR-FFC targeting a TF was the most overrepresented (*P *≈ 0.060, see the Methods) among the FFCs that target TFs.

The aspect of these higher-order DAR-FFCs provides another interesting network feature. Among the five higher-order DAR-FFCs (Figure 3), KLF12 is the only node that always acts as an originating regulator in all three higher-order DAR-FFCs in which it is involved. This TF (KLF12) shows a clear contrast to ZBTB7A and GFI1B that have different roles, *i.e*. an originating regulator and two intermediary regulators, and an originating regulator and a target, respectively. These findings suggest the possibility that KLF12 has the distinctive function of being a 'higher-order originating regulator hub' that potentially spreads regulatory effects through DAR-FFC-mediated transmissions. The delineation of the DAR-FFC as a network motif provides further potential to reveal such complexity of the higher-order features of regulatory networks.

### The confidence of the results is robust against TF mode perturbation

**Figure 4:**
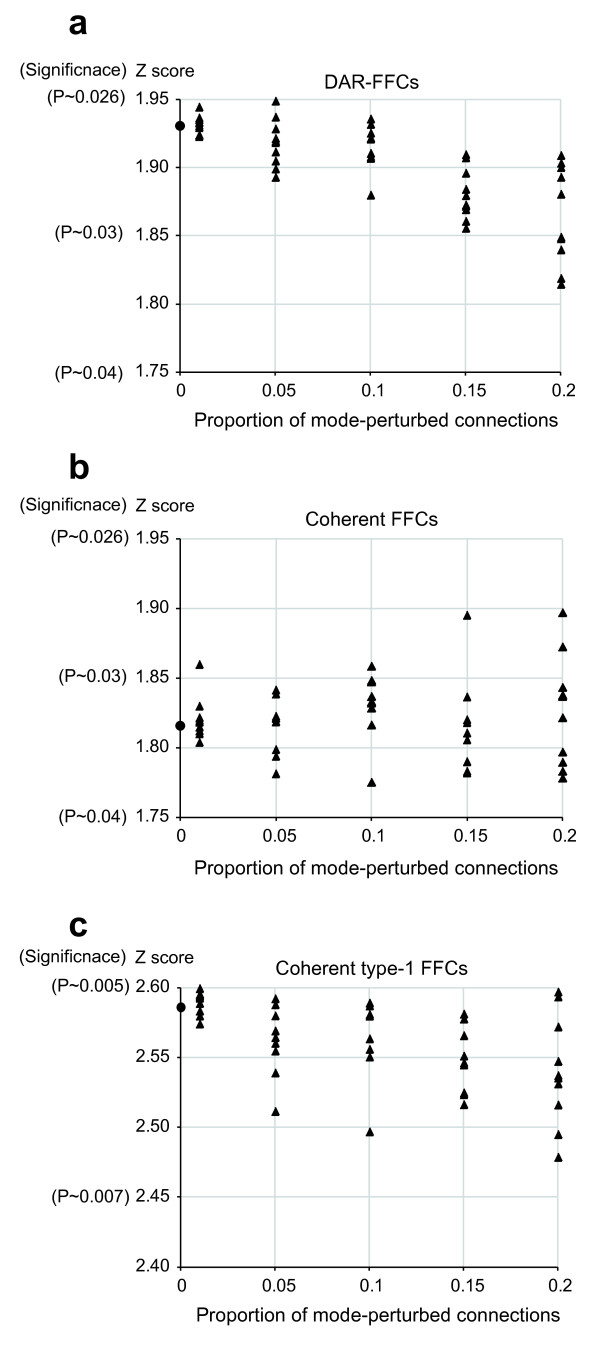
**The changes in Z scores for the overrepresented motifs under assumption of perturbed regulator modes**. The changes in the Z scores are shown in plots with closed triangles along the proportions with which the TF modes of the connections were randomly swapped between positive and negative. This perturbation procedure was repeated ten times with each proportion of 1%, 5%, 10%, 15% and 20% for (a) DAR-FFCs, (b) coherent FFCs and (c) coherent type-1 FFCs. The results show that the Z scores remain at statistically significant levels with mode perturbation of 1% through 20%. The closed circle on each y-axis represents the Z score of the main results (*i.e*. without perturbation).

**Figure 5:**
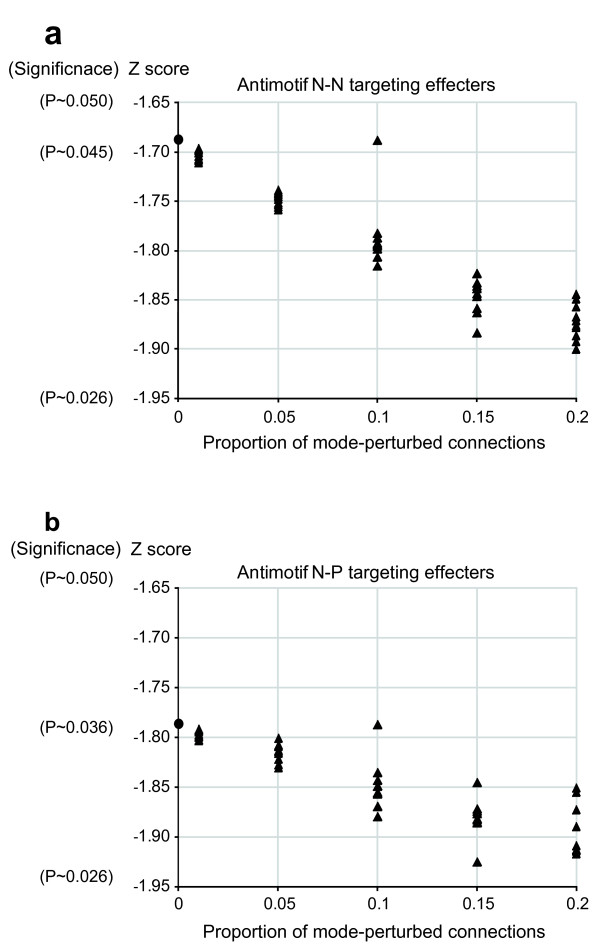
**The changes in Z scores for the antimotifs under assumption of perturbed regulator modes**. The changes in the Z scores are shown in plots with closed triangles along the proportions with which the TF modes of the connections were randomly swapped between positive and negative. This perturbation procedure was repeated ten times with each proportion of 1%, 5%, 10%, 15% and 20% for (a) antimotif N-N targeting effecters, *i.e*. effecter-targeting FFCs composed of an originating repressor regulator with autoregulation and an intermediary repressor regulator without autoregulation, and (b) antimotif N-P targeting effectors, *i.e*. effecter-targeting FFCs composed of an originating repressor regulator with autoregulation and an intermediary activator regulator without autoregulation. The plots show that the confidence levels represented by the Z scores increase despite the mode perturbation procedure. This seemingly paradoxical phenomenon is attributable to the fact that, even if the mode is perturbed, both the antimotif N-N and antimotif N-P targeting effectors come to fall into the category of the originating-only autoregulated FFCs targeting effecters. Therefore, the mode perturbation procedures move the confidence levels for these FFCs closer to the confidence level of the category of the originating-only autoregulated FFCs targeting effecters, the Z score of which is -1.911 (see Figure 1b). The closed circle on each y-axis represents the Z score of the main results (*i.e*. without perturbation).

Since the mode (positive or negative) is not guaranteed for all of the predicted connections regulated by the positive and negative TFs, it is important to examine the robustness of the confidence of the results under the assumption that positive TFs partially act as negative TFs and *vice versa*. For this purpose, we performed mode perturbation analyses by swapping the modes of the connections regulated by positive and negative TFs with proportions of 1%, 5%, 10%, 15% and 20%. As a result, we confirmed that the Z scores that were statistically significant in the results remained at statistically significant levels after the positive-negative mode perturbations of 1% through 20% (Figures 4 and 5). The changes in the Z scores were seemingly paradoxical for the antimotifs (Figure 5), *i.e*. the effecter-targeting FFCs composed of an originating repressor regulator with autoregulation and an intermediary regulator without autoregulation irrespective of the mode, because these confidence levels increased despite the perturbation procedures. This phenomenon is considered to be consistent with the main results based on the following reasoning. Regardless of which originating and intermediary regulators are perturbed to positive or negative, these perturbed FFCs come to fall into the category of the originating-only autoregulated FFCs regardless of the mode (Figure 1b). Therefore, the mode perturbation procedures move the confidence levels for these FFCs closer to the confidence level of the category of the originating-only autoregulated FFCs targeting effecters, the Z score of which is -1.911 (Figure 1b). The plots in Figure 5 show that the Z scores for these antimotif FFCs move closer to the value of -1.911 with an increase in the proportion of the mode-perturbed connections. These observations demonstrate that the confidence of the main results is robust against perturbation of the mode assignments of the positive and negative TFs.

## Discussion

We have demonstrated that the DAR-FFC in which the two repressor regulators are both autoregulated is a distinct and significant network motif in the human transcriptional regulatory network. We were able to improve the resolution power for analyzing the network circuitry by devising an autoregulation-based FFC classification and combining it with the mode-based FFC classification. By focusing on the autoregulation-integrated FFC, we demonstrated the analytical relevance of such small-scale integrated circuits that are positioned between elementary circuit units (*e.g*. autoregulation and simple FFCs) and large-scale integrated circuits (*e.g*. 'dense overlapping regulon') [1]. This autoregulation-based classification has an important logical feature in that it merely depends on the circuit structure without introducing any qualitative features (*i.e*. repressor or activator) of the regulator, and it still enabled the delineation of specific antimotifs.

We also showed that the overall FFC, coherent FFC and coherent type-1 FFC appearances were each significantly overrepresented in the human transcriptional regulatory network based on the conventional mode-based classification. These results are consistent with previous findings in *E. coli *and yeast, suggesting strong evolutionary conservation of the usage of FFCs, particularly type-1 coherent FFCs, at the system level.

The autoregulation-and-mode combined classification enabled us to identify two antimotifs that were both composed of an originating repressor regulator with autoregulation and an intermediary regulator without autoregulation irrespective of the mode. This autoregulation configuration is likely to have a specific disadvantage for feedforward synergistic control of the effecter. From the viewpoint of circuit dynamics, negative autoregulation formed by a repressor has been reported to speed up the response of its own regulator [14]. Therefore, the aforementioned antimotifs that each form a circuit of a quick originating regulator aided by a slower modifier (*i.e*. intermediary regulator) seem to be inappropriate for fine-tuning of the target gene, possibly because the modifier is required to monitor the originating regulator at a higher frequency than the switching frequency of the originating regulator.

The combined classification further revealed the DAR-FFC that targets effecter genes as a novel network motif that comprises two repressors with autoregulation. The overrepresentation of the number of DAR-FFC appearances is possibly explained by the facts that the autorepression integrated in the DAR-FFC provides robustness against stochastic perturbation [15] and accelerates reaching a stable transcription level [14], which consequently make DAR-FFCs advantageous for controlling the effecters. This notion is specifically supported by the finding that the most preferred molecular function of the DAR-FFC-targeted effecters was 'growth factor activity' (Table 2), since cellular responses to a growth factor have been reported to require a set of key 'repressive' transcriptional regulators to achieve tightly controlled signal attenuation processes [16]. Consequently, the swiftness and robustness of DAR-FFCs are suitable properties for the signaling systems of various growth factors.

In addition to 'growth factor activity', the effecter functions targeted by the DAR-FFCs demonstrated a marked preference for intercellular communications through humoral, neuronal and ECM-mediated signaling modalities, representing the major intercellular communications in higher eukaryotes. Another functionality significantly preferred by the DAR-FFC-targeted effecters included transcription-modifying activities that increase the information integration capacities at the transcriptional level. These properties of DAR-FFCs have a crucial advantage for enhancing the information transmission and integration that is inevitable for the inflated needs of information processing that are possibly brought about multicellularization.

Notably, and consistent with the above notion, we found that 'multicellular organismal development' was by far the most preferred biological process among DAR-FFC-targeted effecters. It is also noteworthy that 'multicellular organismal development' was followed in the list by biological processes related to the neuronal cell fate program, namely 'nervous system development', 'Wnt receptor signaling pathway' and 'axon guidance'. This preference for nervous system development is a general feature of DAR-FFCs because all the individual DAR-FFC connections identified in the present study had effecter genes with preferred functions within 'multicellular organismal development' and 'nervous system development'. Furthermore, our network analyses revealed that the DAR-FFCs were densely interconnected, and that individual DAR-FFCs formed a higher-order DAR-FFC topology. This topology included a possible 'higher-order originating regulator hub' that potentially spreads regulatory effects through DAR-FFC-mediated transmissions. These dense interlinking features of DAR-FFCs are likely to provide further robustness for transcriptional regulatory networks involved in multicellularity and nervous system development.

Based on the results of the network motif representation analyses, GO functional analyses and higher-order network structures, we suggest that the DAR-FFC identified in the present study is a distinctive integrated network motif endowed with properties that are indispensable for forming the transcriptional regulatory circuits essential for multicellular organization and nervous system development. An intelligible and comprehensive description of the transcriptional regulatory system requires an appropriate set of network motifs that would include integrated network motifs such as the DAR-FFC. It is necessary to elucidate other potential integrated network motifs with a sufficient descriptive power for understanding the gene regulatory system. The degree of integration or abstraction of these motifs would vary depending on the purpose of the description of the system.

## Conclusions

We have demonstrated that the configuration of autoregulation integrated in the FFC critically affects the FFC abundance or avoidance. In particular, we found that the DAR-FFC composed of two repressors with autoregulation is a novel significantly overrepresented network motif. Notably, we have revealed that DAR-FFCs constitute transcriptional circuits that preferentially control multicellular organization and nervous system development. These results suggest that the DAR-FFC is a key component that is essential for the multicellularization of higher eukaryotes.

## Methods

### Human-mouse orthologous upstream sequences

We downloaded genome sequences and annotations for humans (Build 37.1) and mice (Build 36.3) from NCBI at http://ftp.ncbi.nih.gov/genomes/. We parsed the annotations and selected protein-coding genes that did not overlap with other genes. We further chose genes whose upstream sequences did not overlap with other genes for at least 8 kb upstream of the translation start site. If a gene had alternative transcription/translation start or end sites, we always adopted the 5'-most site for the start site and the 3'-most for the end site. These retrieval processes yielded 14021 genes for humans and 16412 genes for mice. To stringently identify human-mouse orthologs, we selected genes whose official gene symbols were identical between humans and mice. This ortholog assignment approach is effective for human-mouse comparisons [17,18], because the same official gene symbol is endowed based on well-curated functional experimental evidence. Finally, we obtained 5169 orthologous 8-kb upstream sequence pairs (Additional file 2 and our website [19]).

### TF gene selection

Among the 5169 orthologs, we identified TF genes according to the GO 'molecular functions' category by surveying the gene2go file of NCBI reference sequences (RefSeq) [20] at http://ftp.ncbi.nlm.nih.gov/gene/DATA/. We regarded genes assigned to 'transcription factor activity' (GO:0003700) as TF genes, which amounted to 307 TF genes. We further selected the TF genes with known binding motifs by searching TRANSFAC [21] Professional 12.2 for the binding motif matrices under the conditions that (I) an official gene symbol of the TF was unequivocally designated, (II) the TF gene was included in the 5169 orthologs, (III) the TF was able to function even if it did not form a complex with other TFs, (IV) the motif matrix was not a composite one with other kinds of TFs, and (V) the motif matrix existed in humans or mice. A total of 82 TFs were assigned to corresponding motif matrices that fulfilled the above five conditions. To identify the mode of each of the 82 TFs, *i.e*. positive (activator), negative (repressor) or bimodal regulator, we surveyed the descriptions of GO, Entrez Gene [22] and TRANSFAC (Additional file 3).

In the analyses in which the TF mode affected the results, we excluded the counts of every circuit that included a bimodal TF or a mode-unspecified TF, since the particular connections and the proportion of connections that were positive or negative for these TFs were mostly uncertain with regard to our predicted regulatory connections. On the contrary, in cases where the TF mode did not affect the results, the counts of the circuits that included a bimodal or a mode-unspecified TF were not excluded from the statistics. These cases were the analyses of the overall FFCs, the overall autoregulations, and the autoregulation-configuration classification regardless of the mode (Figure 1b).

### TFBS prediction

We searched the 8-kb upstream sequence of each of the 82 TFs for TFBS motifs using the MATCH™ program [23] with a score cutoff of 0.9 and a motif-core score cutoff of 0.8. To identify conserved TFBS motifs, we performed a genomic alignment for each of the 5169 8-kb orthologous upstream sequence pairs using ReAlignerV [18] with its default settings. We obtained 76600 conserved blocks whose identities were ≥70%. Next, we retrieved TFBSs that met the following three conditions between the human and mouse motifs: (I) the motif directions were the same; (II) both motifs were located within a conserved block; and (III) the identity of the pair of motifs was ≥90%. Finally, we obtained 386237 conserved TFBSs (Additional file 4 and our website [19]).

### Connection matrix randomization

Transcriptional targeting of the 82 TFs to the 5169 genes was represented as a connection matrix, *M*, in which *Mij *= 1 if TF *j *targets gene *i *and *Mij *= 0 in the absence of such targeting (Additional file 5). Since all of the 82 TF genes were included in the 5169 genes, it was possible to examine autoregulation for the 82 TFs. Starting from the original connection matrix of the real data, we created randomized matrices, so that every TF *j *should target the same number of genes as in the original matrix and every gene *i *should be targeted by the same number of TFs as in the original matrix [1-4]. According to a previously described method [12], we performed the randomization using stepwise processes as follows. We randomly chose a pair of columns (TFs), *a *and *b *(*a *≠ *b*), and a pair of rows (genes), *m *and *n *(*m *≠ *n*). When *M_ma _*= 1 and *M_nb _*= 1, we further checked whether *M_na _*= 0 and *M_mb _*= 0. If the four equations were satisfied, we swapped the elements such that *M_ma _*= 0, *M_nb _*= 0, *M_na _*= 1 and *M_mb _*= 1. We obtained 1000 randomized connection matrices by repeating these swapping steps 500000 times for each randomization.

To determine the number of swapping steps that resulted in sufficient randomization of the original connection matrix, we monitored the number of value-changed elements from the original connection matrix by varying the number of swapping steps (100, 1000, 10000, 50000, 100000, 500000, 1000000 and 5000000 steps), and for each number of swapping steps, we repeated the randomization 100 times. As a result, the number of value-changed elements reached a plateau phase with 500000 swapping steps, and the use of 1000000 and 5000000 swapping steps yielded no significant increases in the numbers of value-changed elements. Therefore, we determined that 500000 swapping steps were sufficient for randomization of the original connection matrix.

### GO enrichment analyses

First, we examined the background set of GO 'molecular functions' terms for the 4862 effecter genes (*i.e*. 5169 total genes - 307 TF genes). The total number of redundant appearances of the GO terms assigned to all the effecters was denoted as *E *(*E *= 12319), and the number of non-redundant appearances of the GO terms was denoted as *N *(*N *= 1562). For each non-redundant GO term *g *of the *N *terms, the number of appearances within all the effecters was denoted as *F_g_*.

Next, we focused on the DAR-FFC-targeted effecters. The total number of redundant appearances of the GO terms of all the DAR-FFC-targeted effecters was denoted as *D *(*D *= 31105). The number of appearances of each *g *for all the DAR-FFC-targeted effecters was denoted as *f_g_*. It should be noted that there were cases in which an effecter was targeted by multiple DAR-FFCs. The probability of *g *appearing in the background set was denoted as *r_g_*, which was computed as *r_g _*= *F_g _*/*E*. For each *g*, we assumed that *f_g _*followed a binomial distribution with a probability *r_g _*and number of trials *D*. Therefore, for each *g*, we computed the probability *P_g _*of the *g *appearing more than *f_g _*times in all the DAR-FFCs as follows.(1)

We ranked the *P_g _*values in increasing order to show the highly enriched GO terms. The GO terms that appeared at frequencies of ≤0.1% of *E *were discarded from the list. We separately conducted the same procedures described above for the GO 'biological processes' terms.

We performed GO 'biological processes' enrichment analyses for each of the 13 DAR-FFC TF-TF connections separately. The total number of redundant appearances of the GO terms assigned to effecters targeted by the *i*-th DAR-FFC TF-TF connection was denoted as *D_i_*. The number of appearances of each *g *for the effecters targeted by the *i*-th DAR-FFC TF-TF connection was denoted as *f_(g,i)_*. We used the same value *r_g _*as above. For each *g *and each *i*, we computed the probability *P_(g, i) _*as follows.(2)

The GO terms with frequencies of ≤0.05% of *E *or *P_(g, i) _*values of ≥0.01 were discarded from the list for these analyses.

### Statistical analyses

For each circuit, the mean and standard deviation were computed based on the appearance in each of the 1000 randomized matrices, and the *P *value and Z score were estimated by assuming a standard normal distribution.

### TF mode perturbation analyses

To assess the robustness against perturbation of the mode assignments of the TFs, we randomly selected the connections regulated by positive TFs and negative TFs with proportions of 1%, 5%, 10%, 15% and 20%, and stored the selected connections for each proportion. This procedure was repeated 10 times for each of the five proportions. To the selected connections stored for the resulting 50 sets, we applied swapped modes between positive and negative, and subsequently computed the Z scores based on the 1000 randomized and one real connection matrices that were used in the main analyses.

## List of abbreviations

FFC: feedforward circuit; DAR-FFC: double-autorepression feedforward circuit; TF: transcription factor; TFBS: transcription factor binding site; GO: Gene Ontology; ECM: extracellular matrix; NCBI: National Center for Biotechnology Information; RefSeq: reference sequence.

## Authors' contributions

H Iwama, KM and TI jointly designed the study. H Imachi, KM and H Iwama scrutinized the TF annotations. H Iwama wrote computer scripts and the manuscript. All the authors discussed the results, commented on the manuscript and approved the final manuscript.

## Supplementary Material

Additional file 1 **Preferred biological processes of the effecter genes targeted by each of the 13 TF-TF connections of DAR-FFCs**. The overrepresented GO terms for biological processes are listed for each of the 13 TF-TF connections of DAR-FFCs. Click here for file

Additional file 2 **Genomic nucleotide sequences 8-kb upstream of the translation start site for 5169 pairs of human-mouse orthologs**. The genomic nucleotide sequences 8-kb upstream of the translation start site are shown for 5169 pairs of human-mouse orthologs together with the corresponding gene symbols and gene IDs. Click here for file

Additional file 3 **Regulator mode annotations of the 82 TFs used to identify the TFBSs**. The annotations of the regulator modes (repressor, activator or bimodal) are shown for the 82 TFs used in this study together with the Gene IDs and the annotation source. Click here for file

Additional file 4 **Alignments of conserved blocks for 5169 human-mouse orthologs with conserved TFBSs**. The location and motif information for 386237 TFBSs are displayed in a character-based alignment-embedded layout. Each file name stands for a gene symbol and the file contains the alignments of conserved blocks with TFBS information for the corresponding gene. Each alignment is not interleaved. The graphical alignments with TFBS information for the 5169 human-mouse ortholog pairs are provided in a browsable format at our website http://genet.med.kagawa-u.ac.jp/pub/HMO_5169/List.htm, together with the character-based alignments with TFBSs and raw sequences contained in Additional files 4 and 2 respectively. Click here for file

Additional file 5 **Transcriptional connection matrix *M***. The transcriptional targeting of the 82 TFs to the 5169 genes is shown as a matrix *M *in which *Mij *= 1 if TF *j *targets gene *i *and *Mij *= 0 in the absence of such targeting. Each TF *i *is designated with an official gene symbol in a column and each target gene *j *is designated by an NCBI Entrez Gene ID. Click here for file
