# Supplementary material for: Transcriptional double-autorepression feedforward circuits act for multicellularity and nervous system development
Source: BMC Genomics. 2011 May 11;12:228. doi: 10.1186/1471-2164-12-228 (PMC3116505; doi:10.1186/1471-2164-12-228)
Supplement: Additional file 1 — Preferred biological processes of the effecter genes targeted by each of the 13 TF-TF connections of DAR-FFCs. The overrepresented GO terms for biological processes are listed for each of the 13 TF-TF connections of DAR-FFCs. [file 1471-2164-12-228-S1.PDF]

Additional file 1

Preferred biological processes of the effecter genes targeted by each of the 13 TF-TF connections of DAR-FFCs

(Please see color-coding chart at the bottom of the table)

ZBTB7A--E2F1

multicellular organismal development	2.0E−05
axon guidance	8.4E−04
regulation of transcription, DNA-dependent	2.2E−03
Wnt receptor signaling pathway	2.7E−03
transmembrane receptor protein tyrosine kinase signaling pathway	3.4E−03
cell fate commitment	5.8E−03
negative regulation of Wnt receptor signaling pathway	5.8E−03
regulation of signal transduction	5.8E−03
nervous system development	6.8E−03
Wnt receptor signaling pathway, calcium modulating pathway	7.6E−03
negative regulation of transcription from RNA polymerase II promoter	9.7E−03
chromatin assembly or disassembly	1.0E−02

ZBTB7A--MSX1

multicellular organismal development	1.3E−04
neuron differentiation	2.5E−03
axon guidance	3.1E−03
cell fate commitment	5.9E−03
neuron migration	7.3E−03
potassium ion transport	7.5E−03

ZBTB7A--PRDM1

multicellular organismal development	3.4E−07
regulation of transcription, DNA-dependent	6.4E−03
nervous system development	1.7E−05
Wnt receptor signaling pathway	6.6E−04
angiogenesis	1.5E−04
regulation of transcription from RNA polymerase II promoter	8.8E−03
positive regulation of transcription from RNA polymerase II promoter	7.2E−03
cell-cell adhesion	3.2E−03
heart development	9.6E−03
axon guidance	5.4E−03
regulation of cell migration	4.3E−03
JNK cascade	6.6E−03
mitochondrial transport	6.6E−03
cell fate commitment	3.9E−03
growth	3.9E−03
negative regulation of endothelial cell proliferation	3.9E−03

MSX1--PRDM1

multicellular organismal development	1.4E−06
regulation of transcription, DNA-dependent	9.1E−04
cell adhesion	2.4E−03
Wnt receptor signaling pathway	4.4E−03
angiogenesis	2.8E−04
positive regulation of transcription from RNA polymerase II promoter	9.0E−03
muscle development	1.8E−03
axon guidance	9.0E−03
chromatin assembly or disassembly	2.5E−03
B cell differentiation	7.2E−03
neuron differentiation	4.8E−03
striated muscle contraction	4.8E−03
cell fate commitment	1.7E−03
growth	1.7E−03
regulation of the force of heart contraction	1.7E−03

GFI1B--PRDM1

multicellular organismal development	8.4E−08
B cell differentiation	8.3E−05
Wnt receptor signaling pathway	4.7E−04
nervous system development	4.9E−04
positive regulation of transcription from RNA polymerase II promoter	5.8E−04
angiogenesis	1.9E−03
neuron migration	3.7E−03
regulation of transcription, DNA-dependent	5.2E−03
axon guidance	6.1E−03
Wnt receptor signaling pathway, calcium modulating pathway	6.1E−03
negative regulation of apoptosis	6.3E−03
neuron differentiation	6.9E−03
striated muscle contraction	6.9E−03
response to drug	9.4E−03
response to peptide hormone stimulus	9.5E−03

HES1--GFI1B

multicellular organismal development	1.7E−04
regulation of transcription, DNA-dependent	1.2E−03
nervous system development	6.2E−03
potassium ion transport	4.3E−03
integrin-mediated signaling pathway	9.0E−03
cytoskeleton organization and biogenesis	9.0E−03
collagen fibril organization	6.5E−03
negative regulation of endothelial cell proliferation	3.8E−03

KLF12--CEBPG

homophilic cell adhesion	4.0E−03
regulation of transcription	6.8E−03
regulation of cell migration	8.8E−03
hemopoiesis	8.8E−03

KLF12--E2F1

multicellular organismal development	8.9E−05
axon guidance	1.5E−03
organ morphogenesis	2.4E−03
regulation of transcription, DNA-dependent	2.6E−03
anterior/posterior pattern formation	4.4E−03
nervous system development	5.5E−03
Wnt receptor signaling pathway	5.8E−03
transmembrane receptor protein tyrosine kinase signaling pathway	6.8E−03
brain development	7.8E−03
cell fate commitment	8.1E−03
negative regulation of Wnt receptor signaling pathway	8.1E−03
mRNA export from nucleus	8.8E−03
regulation of signal transduction	8.8E−03

KLF12--GFI1B

homophilic cell adhesion	1.7E−03
collagen catabolic process	2.1E−03
potassium ion transport	3.4E−03
cell adhesion	4.4E−03
regulation of pH	5.2E−03
multicellular organismal development	7.8E−03

GFI1B--MSX1

homophilic cell adhesion	3.2E−04
regulation of pH	1.4E−03
potassium ion transport	1.7E−03
regulation of the force of heart contraction	2.2E−03
ossification	2.5E−03
multicellular organismal development	3.3E−03
cell adhesion	9.2E−03

KLF12--HES1

regulation of transcription, DNA-dependent	1.3E−04
nervous system development	1.3E−04
multicellular organismal development	2.3E−04
Wnt receptor signaling pathway	8.0E−04
potassium ion transport	1.7E−03
transcription	2.9E−03
negative regulation of transcription, DNA-dependent	5.1E−03
cell fate commitment	9.1E−03
mRNA splice site selection	9.1E−03
negative regulation of endothelial cell proliferation	9.1E−03

KLF12--ZBTB7A

multicellular organismal development	1.8E−03
transmembrane receptor protein tyrosine kinase signaling pathway	6.4E−03
nervous system development	9.8E−03

ZBTB7A--CEBPG

multicellular organismal development	1.5E−03
nervous system development	5.9E−03
positive regulation of cell proliferation	4.8E−03
Wnt receptor signaling pathway	2.6E−03
regulation of cell migration	3.1E−03
hemopoiesis	3.1E−03

Color coding

multicellular organismal development<br>cell fate commitment	Multicellularity
nervous system development<br>brain development<br>axon guidance<br>neuron differentiation<br>neuron migration	Nervous system development
Wnt receptor signaling pathway<br>Wnt receptor signaling pathway, calcium modulating pathway<br>negative regulation of Wnt receptor signaling pathway	Wnt pathway
regulation of transcription, DNA-dependent<br>positive regulation of transcription from RNA polymerase II promoter<br>regulation of transcription<br>negative regulation of transcription, DNA-dependent<br>negative regulation of transcription from RNA polymerase II promoter<br>regulation of transcription from RNA polymerase II promoter	Auxiliary transcriptional modification
homophilic cell adhesion<br>cell adhesion<br>cell-cell adhesion	Cell adhesion
