# Supplementary material for: Transcriptional double-autorepression feedforward circuits act for multicellularity and nervous system development
Source: BMC Genomics. 2011 May 11;12:228. doi: 10.1186/1471-2164-12-228 (PMC3116505; doi:10.1186/1471-2164-12-228)
Supplement: Additional file 3 — Regulator mode annotations of the 82 TFs used to identify the TFBSs. The annotations of the regulator modes (repressor, activator or bimodal) are shown for the 82 TFs used in this study together with the Gene IDs and the annotation source. [file 1471-2164-12-228-S3.PDF]

Additional file 3. Regulator mode annotations of 82 TFs that were used to identify the TFBSs

Gene ID	Official gene symbol	Mode (neg: repressor; pos: activator; neg&pos: bimodal)	Annotation sources (TF: TRANSFAC; GO: Gene Ontology)	References
1054	CEBPG	neg	TF, GO	
1869	E2F1	neg	GO	
8328	GFI1B	neg	TF, GO	
3280	HES1	neg	TF, GO	
11278	KLF12	neg	TF, GO	
4487	MSX1	neg	TF, GO	
4824	NKX3-1	neg	TF (ref)	DNA-binding sequence of the human prostate-specific homeodomain protein NKX3.1. Steadman DJ, Giuffrida D, Gelmann EP (2000) *Nucleic Acids Res* 28(12):2389-2395.
639	PRDM1	neg	TF, GO	
7421	VDR	neg	GO	
7704	ZBTB16	neg	TF (mouse), GO	
51341	ZBTB7A	neg	GO	
2019	EN1	neg	ref	Engrailed-1 negatively regulates beta-catenin transcriptional activity by destabilizing beta-catenin via a glycogen synthase kinase-3beta-independent pathway. Bachar-Dahan L, Goltzmann J, Yaniv A, Gazit A (2006) *Mol Biol Cell* 17(6):2572-2580.
367	AR	pos	TF	
466	ATF1	pos	TF, GO	

1386	ATF2	pos	GO	
467	ATF3	pos	GO	
1051	CEBPB	pos	TF	
1385	CREB1	pos	TF	
1406	CRX	pos	TF	
1877	E4F1	pos	GO	
1959	EGR2	pos	GO	
2002	ELK1	pos	TF, GO	
2033	EP300	pos	GO	
2113	ETS1	pos	GO	
2114	ETS2	pos	TF, GO	
2118	ETV4	pos	TF	
2299	FOXI1	pos	TF (ref)	The winged helix transcriptional activator HFH-3 is expressed in the distal tubules of embryonic and adult mouse kidney. Overdier DG, Ye H, Peterson RS, Clevidence DE, Costa RH (1997) *J Biol Chem* 272(21):13725-13730.
2623	GATA1	pos	TF, GO	
2624	GATA2	pos	TF, GO	
2969	GTF2I	pos	GO	
3131	HLF	pos	TF, GO	
3298	HSF2	pos	TF	
22807	IKZF2	pos	TF, GO	
3659	IRF1	pos	TF, GO	
4602	MYB	pos	TF, GO	
4654	MYOD1	pos	TF, GO	
4656	MYOG	pos	TF, GO	
4778	NFE2	pos	TF, GO	
4790	NFKB1	pos	TF, GO	
2516	NR5A1	pos	TF, GO	
2494	NR5A2	pos	TF	
5078	PAX4	pos	TF, GO	
3651	PDX1	pos	TF, GO	

5241	PGR	pos	TF
5308	PITX2	pos	TF, GO
5449	POU1F1	pos	TF, GO
5453	POU3F1	pos	TF, GO
5463	POU6F1	pos	TF
5966	REL	pos	TF, GO
6239	RREB1	pos	TF, GO
4089	SMAD4	pos	TF, GO
64321	SOX17	pos	TF (mouse)
6660	SOX5	pos	GO
6722	SRF	pos	TF, GO
6772	STAT1	pos	TF
6776	STAT5A	pos	TF, GO
6777	STAT5B	pos	TF, GO
6862	T	pos	TF (mouse), GO (mouse)
6929	TCF3	pos	TF (mouse), GO
6925	TCF4	pos	TF, GO
7003	TEAD1	pos	GO
1958	EGR1	pos & neg	TF, GO
2099	ESR1	pos & neg	GO
3660	IRF2	pos & neg	TF (pos), GO (neg)
3394	IRF8	pos & neg	TF (pos&neg), GO (neg)
4609	MYC	pos & neg	GO
1482	NKX2-5	pos & neg	TF (pos), GO (pos&neg)
5451	POU2F1	pos & neg	TF, GO
5468	PPARG	pos & neg	GO
6667	SP1	pos & neg	TF
6774	STAT3	pos & neg	TF (pos), GO (neg)
7528	YY1	pos & neg	GO
1761	DMRT1	unspecified	
10655	DMRT2	unspecified	
63951	DMRTA1	unspecified	
84337	ELOF1	unspecified	

2297	FOXD1	unspecified
2300	FOXL1	unspecified
8456	FOXN1	unspecified
51450	PRRX2	unspecified
7494	XBP1	unspecified
51043	ZBTB7B	unspecified
